# Influence of Manufacturing Mechanical and Thermal Histories on Dimensional Stabilities of FR4 Laminate and FR4/Cu-Plated Holes

**DOI:** 10.3390/ma11112114

**Published:** 2018-10-28

**Authors:** Alexandra Rudajevová, Karel Dušek

**Affiliations:** Department of Electrotechnology, Faculty of Electrical Engineering, Czech Technical University in Prague, Technická 2, 166 27, Prague 6, Czech Republic; alex.rud@email.cz

**Keywords:** PCB laminate, thermal analysis, thermomechanical properties, residual/internal stress

## Abstract

Irreversible dimension changes of an FR4 laminate board in the z-direction and FR4 laminate/Cu plated holes that depend on their manufacturing histories have been studied by thermal mechanical analysis in the temperature range from room temperature to 240 °C. It is found that the compression residual stresses generated in both materials due to manufacturing pressing are released during heating, leading to an elongation in the specified direction. This increase depends on the composition of the studied composite and number of pressing cycles. The second reason for the observed dimensional changes is insufficient curing during manufacture that causes post-curing after the first heating cycle and related board shrinkage in the z-direction. The temperature regions of these two processes are not the same. The post-curing process occurs in the transition temperature range (near the glass transition temperature), whereas the release of the compression residual stress is observed at higher temperatures. Both these processes are temperature-dependent and do not proceed to completion during one heating cycle. Moreover, the compression residual stress strongly influences the post-curing process.

## 1. Introduction

The long service life of an electronic assembly is a basic requirement for its manufacturer, and depends on the dimensional stability of the printed circuit board (PCB). A basic PCB is composed of polymer, glass fibers, and Cu conductive material. The combination of these different materials generates various internal stresses in the composite material (even during its manufacture). As a result, their subsequent thermal loading leads to the release of the internal stresses causing dimensional changes of the final product.

FR4 laminate [1] is the most widely used material for the fabrication of PCBs. Its woven glass component acts as a reinforcement for the laminate matrix, and the resin serves as a binder and a load transfer agent. Various curing agents, flame retardants, fillers, and accelerators are added to the resin to tailor the laminate mechanical properties. A multi-layered PCB is prepared by pressing several prepregs and cores together (with etched copper cladding as per circuit requirements) at high temperature and pressure. Through holes and micro-via interconnects are drilled in the produced PCB and then plated with copper. Finally, the board is subjected to reflow soldering to complete the printed circuit assembly.

The soldering of electronic components to the basic board is usually performed in the temperature range from room temperature (RT) to 250 °C (Sn–Cu–Ag solders). Cu exhibits the simplest temperature dependence of its thermal properties in the specified range, whereas Sn solder undergoes melting-solidification phase transitions (this process is related to thermal and undercooling effects). The largest changes of the thermal properties in this temperature range are observed for FR4 laminate, which represents a strongly anisotropic composite with physical properties depending not only on its composition and structure, but also on the thermal and mechanical histories. Furthermore, FR4 laminate is a viscoelastic material [2] and, therefore, exhibits combined elastic and viscous characteristics during heat treatment separated by the glass transition temperature (Tg).

The incorporation of internal Cu layers into the FR4 laminate structure increases the thickness of the basic substrate and generates thermal and residual stresses due to the mismatch between the coefficients of thermal expansion (CTEs) of the utilized components. The relaxation of these stresses leads to dimensional changes of the PCB. Several studies have addressed the shrinkage effect in PCBs observed after a reflow process [3,4,5]. Moreover, studies and reviews published 20 years ago focused on the influence of the material composition, manufacturing process, and design parameters on the reliability of plated through holes in multi-layer PCBs [6,7,8]. This problem remains unsolved even today [9,10]. In recent studies, the temperature stabilities of plated through holes, buried holes, and microvias used to interconnect various PCB layers have been examined. Salahouelhadj et al. [9] conducted experimental and theoretical study of the Cu plating thickness, hole diameter, and PCB thickness on reliability. The obtained results confirmed that the difference in CTE along the z-axis of the material was a key parameter for reliability analysis. Rao et al. [10] have reported high-Tg woven fiberglass multifunctional epoxy FR-4 laminates that are now commonly used for PCB fabrication. The higher the Tg of a material, the better is its dimensional stability; in addition, a relatively low CTE allows it to endure multiple soldering operations.

The influence of lead-free soldering on the key material properties of FR4 laminate was studied by Sanapala at al. [11]. They determined the values of Tg, CTE, decomposition temperature, and water absorption capacity for a number of commercially available FR4 laminate materials classified based on Tg as well as on the presence of curing agents, flame retardants, and fillers. The CTE values of the exposed samples were lower than those of the samples before exposure due to the further curing of the epoxy system, which increased its cross-linking density. Post-curing effect in the x-, y-, and z-directions leading to material shrinkage was observed in our previous study on FR4 laminate, which showed that the largest dimensional changes occurred along the z-direction [12].

Many studies have focused on overcoming various problems caused by the difference in the material characteristics of PCB components. New materials with better properties were developed to lower the residual thermal stress. Hart [13] recommended replacing spherically-shaped balls with cylindrically-shaped solder columns to decrease the CTE mismatch during microelectronics assembly (the limitations of this approach were discussed elsewhere [14]). Chung et al. [15] performed thermomechanical analysis of PCBs during the reflow process for warpage prediction. In the work of Weihold and Yen [16], a possible way to minimize the CTE difference between the PCB laminate and package/chip is discussed to improve stress management during electronics assembly. The results of these and other studies show that the problem of different physical properties of PCB materials still remains relevant.

The main reasons for the irreversible dimensional changes of PCBs (which occur during the first thermal loading after manufacture) are the existence of residual stresses in the FR4 laminate structure and post-curing process. Both processes strongly depend on the mechanical and thermal histories of the material (it should be noted that FR4 laminate is a viscoelastic material whose dimensional changes are time-dependent).

A detailed study on the relaxation of residual stresses and post-curing process that occur in FR4 laminate and FR4 laminate/Cu-plated holes has not been performed yet. Thus, the objective of this work was to investigate the residual strain and strain caused by the post-curing effect in FR4 laminates and FR4/Cu holes with various mechanical and thermal histories by performing thermal mechanical analysis (TMA) in the temperature range from RT to 240 °C. As a result, the influence of the mechanical and thermal histories on the FR4 laminate characteristics were determined, and mutual effects of the internal stresses and post-curing process were analyzed.

## 2. Experimental

Samples for analysis were prepared by the Nan Ya company. Their base materials were obtained by pressing two FR4 core layers with one layer of prepreg at a high temperature of about 180 °C. Base material for FR4 with Cu-plated holes was prepared from two layers of FR4 laminate with the Cu foil and prepreg layers using the method for the fabrication of pure FR4. The thickness of Cu foil was 35 µm and the diameter of holes was 1mm. FR4 laminate with Cu was manufactured as described previously by pressing Cu foils/prepreg on the FR4 core. After that, holes were drilled in the resulting composite followed by Cu burning. Finally, Cu holes were fabricated by an electrolytic method.

**Figure 1:**
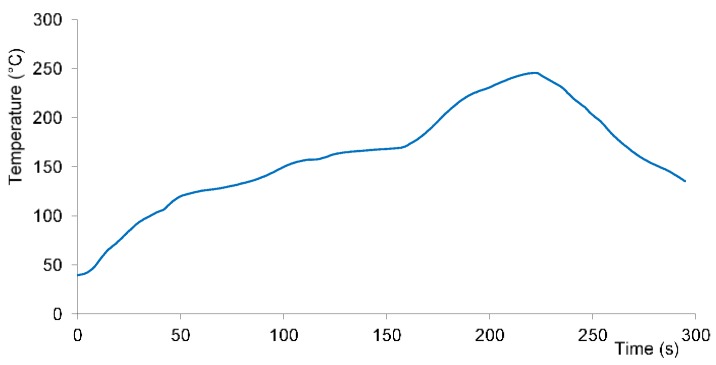
Used temperature profile of reflow oven Mistral 260.

Samples for measuring expansion characteristics had sizes of 5 mm × 5 mm and thicknesses of 1.5 mm. For every experiment minimum three samples were measured. They were studied in the as-prepared state and after the passage through a real reflow oven Mistral 260 (Spidé, Harderwijk, The Netherlands) programmed for the soldering process during the electronic assembly fabrication. Figure 1 shows used temperature profile.

**Figure 2:**
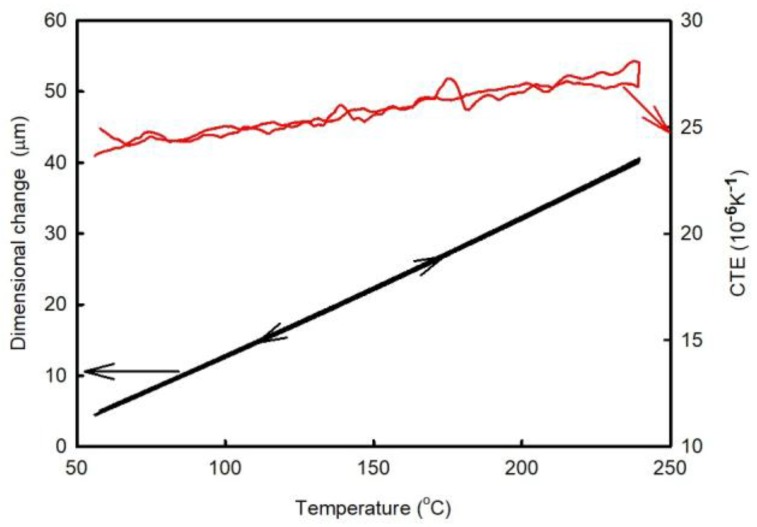
Dilatational characteristics of the calibration Aluminum sample with thickness 7.56 mm. CTE: coefficients of thermal expansion.

Samples of pure FR4 in the as-prepared state were marked as ‘A1’, and after the passage through the oven—as ‘A2’. Samples containing Cu-plated holes in the as-prepared state were marked as ‘B1’, and after their passage through the oven—as ‘B2’. Each type of the marked samples had a different thermal history. Cu-plated holes were drilled in the centers of the B-type samples. Dimensional changes were measured by a TMA Q 400 apparatus (TA Instruments, New Castle, DE, USA) under N_2_ atmosphere at a rate of 2 K/min and probe force of 0.1 N. Two thermal cycles (runs) were performed for each sample in temperature range from RT to 240 °C. Measurement accuracy was verified by determining the thermal expansion characteristics of the pure Al samples (the obtained results are presented in Figure 2). The deviations of the measured CTE values from the tabulated were less than 5%.

## 3. Results and Discussion

The results of previously conducted dilatometry or TMA studies mostly contained only the heating branches of one thermal cycle [17]. However, omitting the cooling parts of the temperature dependences of dimensional changes leads to a loss of important information. The data obtained in this work show that dimensional changes of the studied samples occur during the entire thermal cycle that included both the heating and cooling stages. Dilatometry or TMA experiments typically measure all the length changes that are occurred during the heat treatment of materials (since material strains are additive). Apart from the basic thermal expansion caused by atomic vibrations, many other processes also affect the length changes caused by thermal treatment (both reversible and irreversible ones). 

The mechanical and thermal histories of the A1, A2, B1, and B2 samples are different, as was mentioned in the Experimental section. All the prepared samples represent two-layer FR4 laminates that were subjected to at least one mechanical pressing cycle. This type of preparation can prevent the generation of the residual compression stress and strain in the FR4 laminate structure. The A1 samples underwent only one pressing cycle, whereas the A2 samples were subjected to both mechanical (manufacturing pressing) and heat (inside the Mistral oven) treatments. Hence, samples A1 and A2 differ only by their thermal histories, whereas their mechanical histories are the same. Samples B1 and B2 underwent pressing of the Cu foils/prepreg/FR4 core followed by laminating (pressing) all together and therefore they have the same mechanical history, but different thermal histories. However, the mechanical history of the type A samples is different from that of the samples of type B. Any pressing of materials generates residual stress, which is removed during the first heating cycle. Hence, the first heat treatment cycle for A1 and B1 samples is a measuring in TMA device; for A2 and B2 samples is a heating in the reflow oven Mistral 260. 

**Figure 3:**
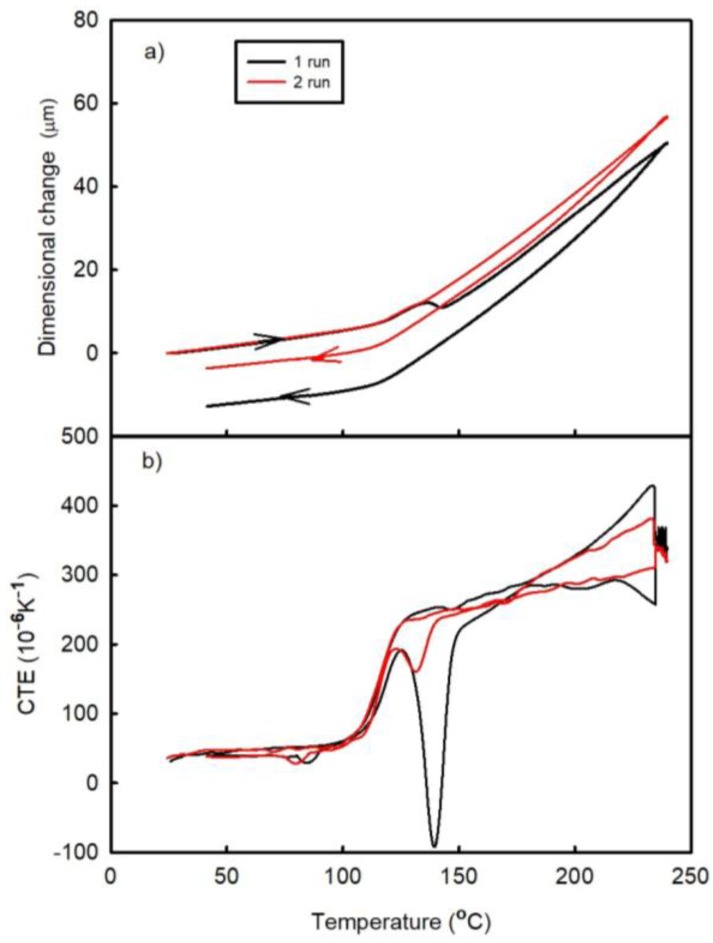
Temperature dependences of the (**a**) dimensional changes and (**b**) CTE of the A1 sample (FR4 laminate) observed after two runs.

**Figure 4:**
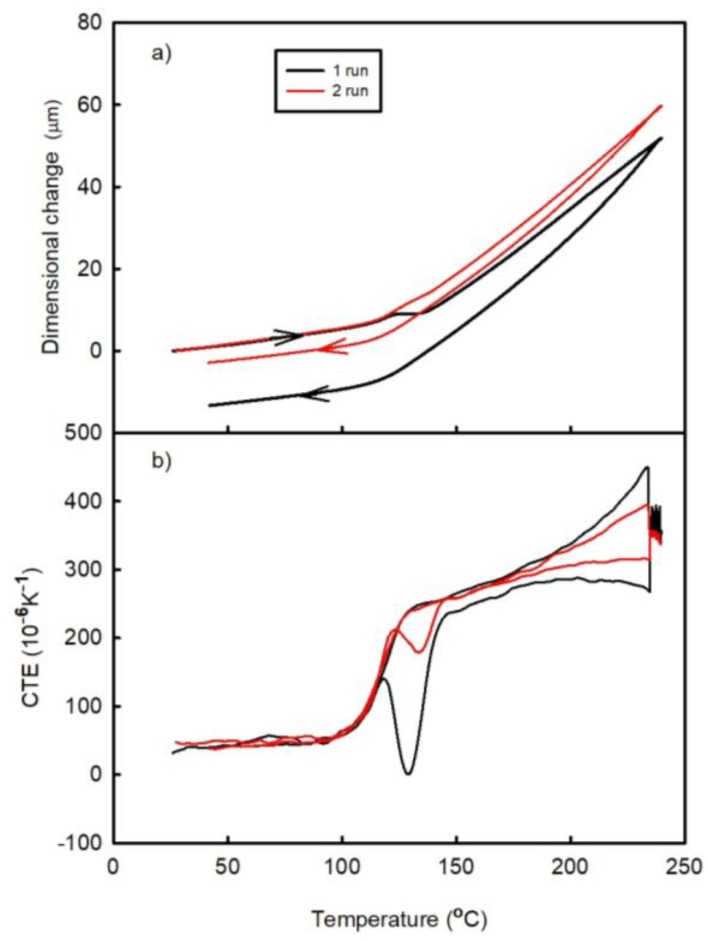
Temperature dependences of the (**a**) dimensional changes and (**b**) CTE of the A2 sample (FR4 laminate passed through the oven) observed after two runs.

Figure 3 and Figure 4 show the temperature dependences of the dimensional changes and CTE, respectively, observed for FR4 laminate in the as prepared state (A1) and after its passage through the oven (A2). Each figure contains the results obtained after two thermal cycles (runs). Both laminates exhibit almost linear dependences of the specified parameters between the lowest temperatures and approximately 100 °C. The temperatures between 100 °C and 150 °C correspond to the transition region between the elastic and viscous states, in which the post-curing process occurs (since the laminate is not fully cured after being received from the manufacturer). The latter produces a negative effect on the dimensional changes (represented by the peak on the temperature dependence of CTE). The post-curing temperature of the A1 laminate sample is greater than that of the A2 material.

During the first thermal cycle, minimum three processes occur in the laminate structure: thermal expansion, post-curing, and release of the manufacturing residual strain. The relationship between these processes and the dimensional changes of the studied sample can be described by the following additive formula:

Δl(t) = ε_B_(t) + ε_C_(t) + ε_P_(t) (1)

where Δl(t) is the total length change of the sample in the z-direction at a given temperature, ε_B_(t) is the strain due to basic thermal expansion, ε_C_(t) is the strain due to the post-curing effect, and ε_P_(t) is the strain due to the release of manufacturing strain. All these variables are time dependent. It can be assumed that both types of samples A1 and A2 have the same first term ε_B_(t) in equation (1). Furthermore, the effects of the post-curing process during the first run are not the same for both materials, as indicated by the areas of the corresponding CTE peaks in the transition range (see Figure 3b and Figure 4b). It can be seen that the post-curing peak on the temperature dependence of the CTE of A1 laminate is larger than that obtained for the A2 laminate sample. A2 laminate was heated during the reflow process in the Mistral 260 oven; hence, its first measuring run corresponded to the second thermal cycle. It can be assumed that part of the curing process took place in the oven during reflow process. The influence of the compression deformation in the z-direction on the temperature dependence of CTE in the high-temperature range (above the temperature of the post-curing process) is represented by a concave curve, which results from the release of the residual compression strain that increases both ε_P_(t) and Δl(t) values. During the second thermal cycle, the temperature dependence of CTE became almost a linear one indicating that the compression residual strain was released during the first run. The main process influencing the irreversible dimensional changes of laminate is the post-curing process, which decreases its dimension in the z-direction. The released amount of the compression residual strain is small as compared to the dimensional changes caused by the post-curing process. Therefore, the irreversible dimensional changes after the thermal cycle have a negative sign.

**Figure 5:**
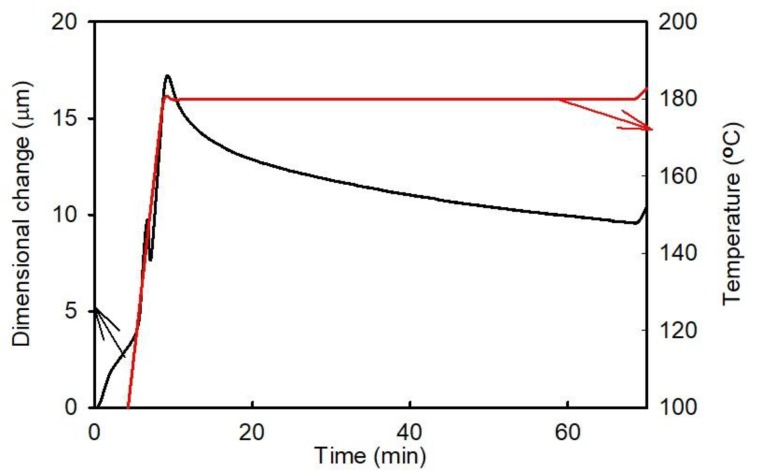
Time dependence of the dimensional changes observed for the A1 sample (FR4 laminate) at 180 °C.

**Figure 6:**
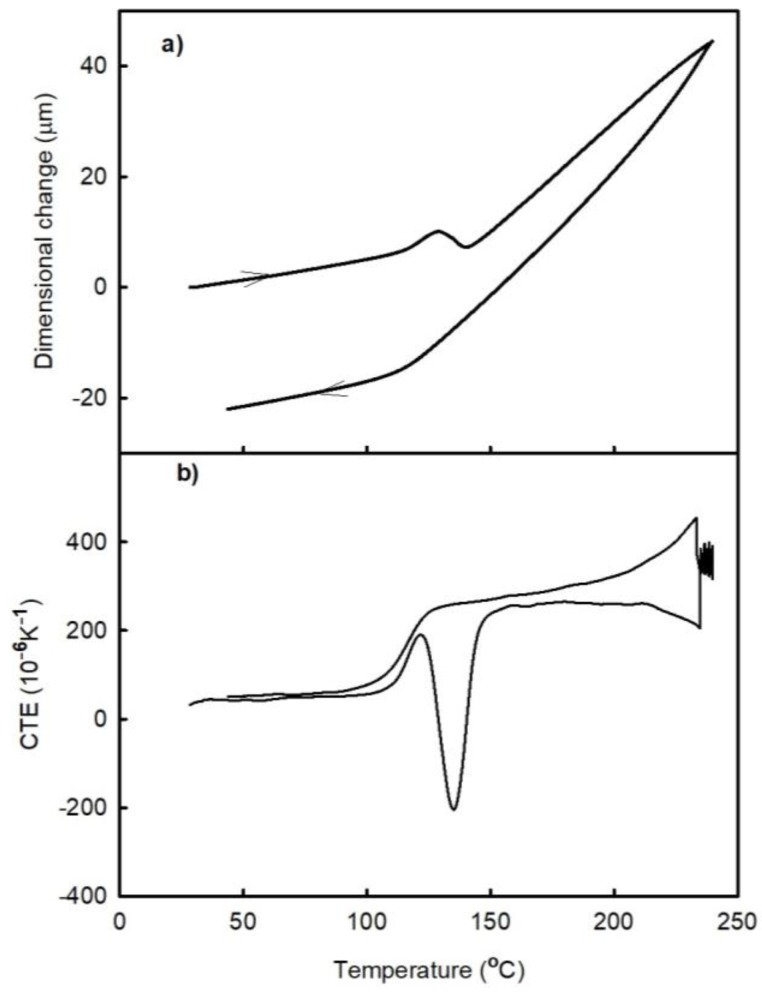
Temperature dependences of the (**a**) dimensional changes and (**b**) CTE of the A1 sample (FR4 laminate) observed after the heat treatment at 180 °C.

The time dependence of dilatational characteristics was determined for the as-prepared A1 sample. First, the A1 laminate was heated to 180 °C at a heating rate of 18 K/min, and the resulting temperature was maintained constant for 1 h. The obtained data are shown in Figure 5. During the isothermal process, the sample dimension decreased over the entire period, which is a consequence of the viscous nature of the laminate. The post-curing process occurs only in a specific temperature range, and the related relaxation of the compression residual strain is small and positive. The results of the second run performed on this sample are presented in Figure 6. It shows that the post-curing process in this experiment did not proceed to completion during the first run characterized by the isothermal segment.

**Figure 7:**
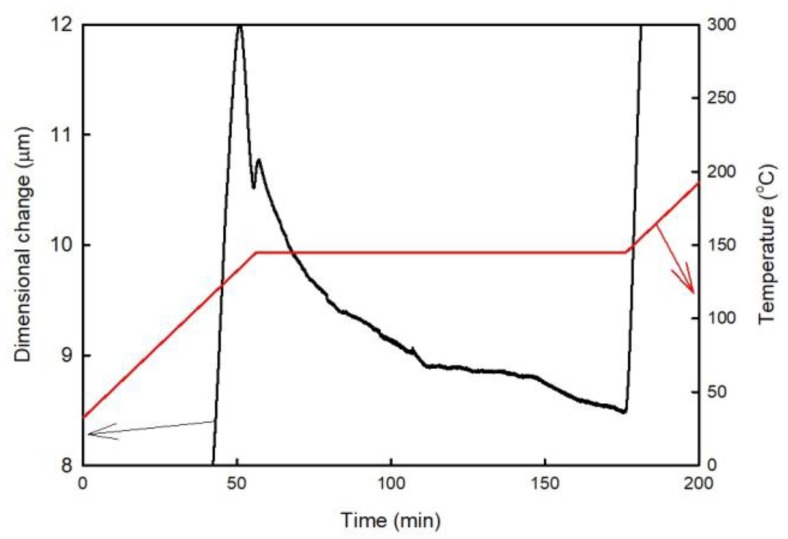
Time dependence of the dimensional changes of the A1 sample (FR4 laminate) observed at 145 °C.

**Figure 8:**
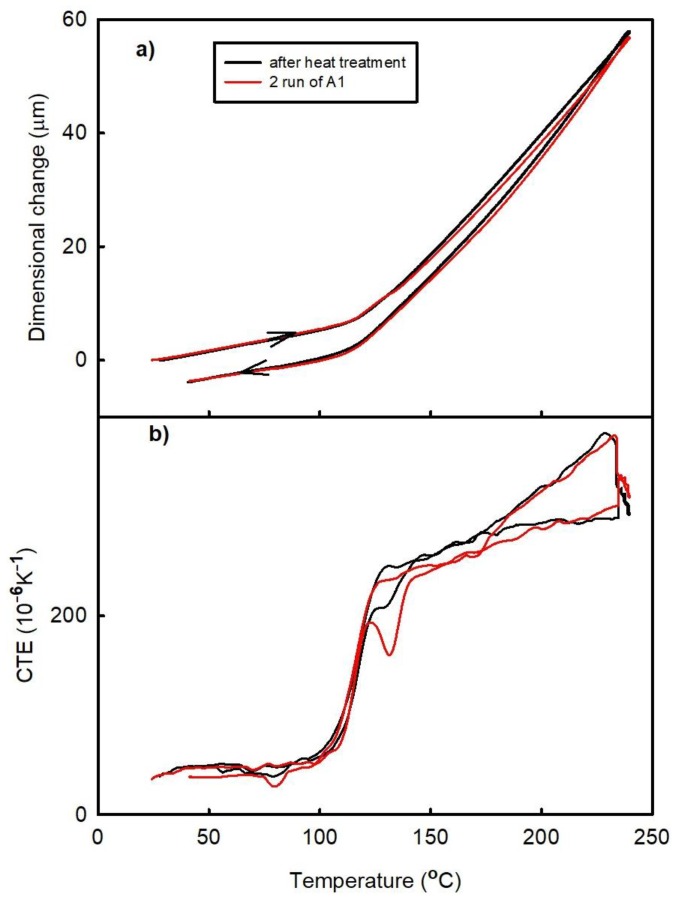
Temperature dependences of the (**a**) dimensional changes and (**b**) CTE of the A1 sample (FR4 laminate) observed after the heat treatment at 145 °C.

The time dependence of the dimensional changes observed during the post-curing process is shown in Figure 7. During this experiment, the temperature was increased to 145 °C at a rate of 2 K/min and then held constant for 2 h. The obtained results reveal that the sample thickness decreased over the entire period. After that, the temperature was increased to 240 °C at a rate of 2 K/min and then cooled at the same rate. After this experiment, an additional thermal cycle was performed at a heating/cooling rate of 2 K/min (the results of these measurements are shown in Figure 8). Figure 8 reveals that the post-curing process took place almost completely during isothermal segment 145 °C. This temperature corresponds to a temperature where the rate of the curing process is high (Figure 2b). The temperature of the peak maximum corresponds to a temperature where the rate of the process is the highest. For comparison, the temperature dependence of the dilatational characteristics obtained for the A1 sample during the second run is presented as well.

**Figure 9:**
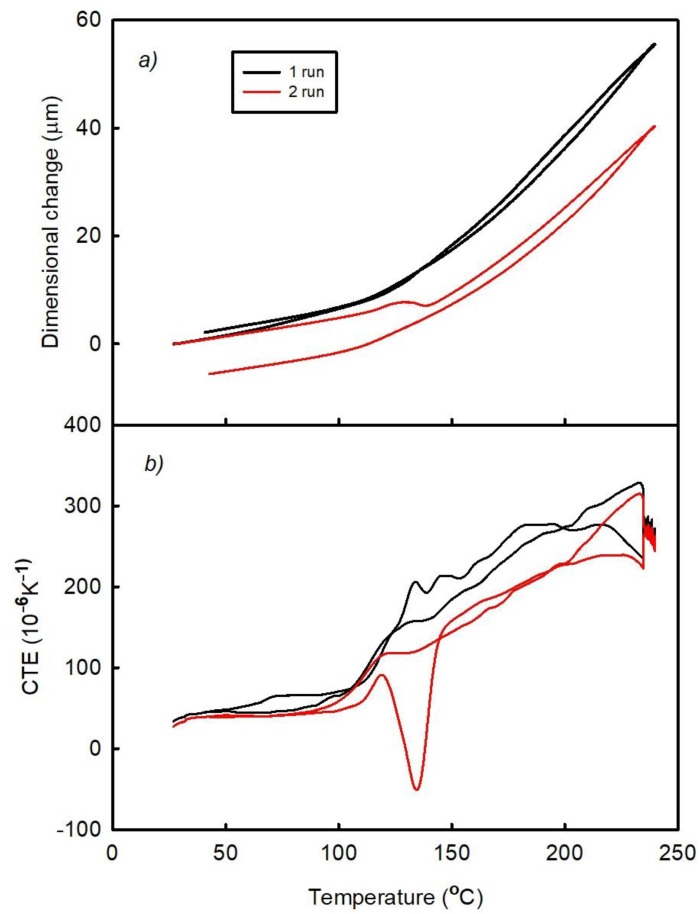
Temperature dependences of the (**a**) dimensional changes and (**b**) CTE of the B1 sample (FR4 laminate/Cu plated holes) observed after two runs.

**Figure 10:**
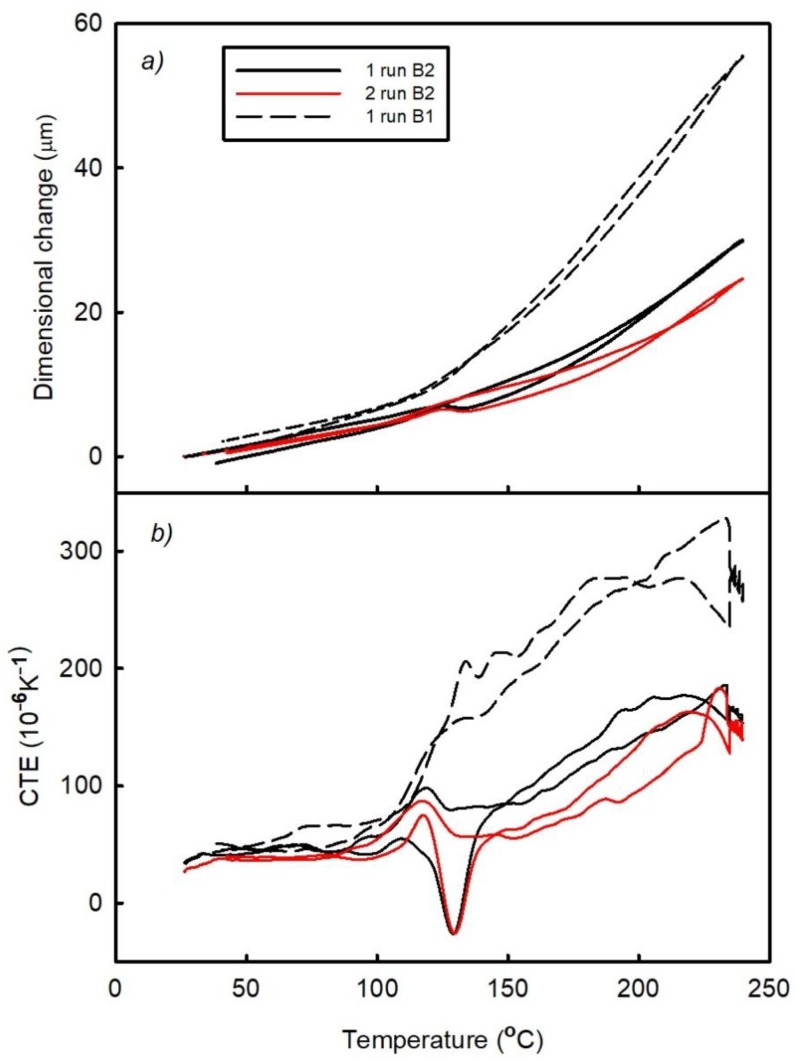
Temperature dependences of the (**a**) dimensional changes and (**b**) CTE of the B2 (FR4 laminate/Cu plated holes) and B1 samples observed after two runs and one run, respectively.

The results obtained for the B1 and B2 samples are shown in Figure 9 and Figure 10, respectively. During measurements, the probe of the TMA instrument was placed on the Cu-plated hole located in center of the sample. This hole was surrounded by FR4 laminate; hence, the thermal expansion of Cu is influenced by the thermal expansion of the laminate. This effect is shown on the temperature dependence of the thermal expansion data obtained between the lowest and the highest temperatures. The trends observed for the temperature dependences of the dimensional changes and CTE of these samples are identical to those obtained for pure FR4 laminate despite placing the TMA probe on the Cu layer. The CTE values of the Cu, A1, and B1 samples measured after 2 runs are listed in Table 1.

The results presented in Table 1 and Figure 9 and Figure 10 clearly show that tensile stress is generated in the Cu holes during the entire thermal cycle. The wall of the Cu hole cannot expand naturally because Cu has a far lower CTE than the laminate in the z-direction. The large tensile stress in the Cu hole can lead to formation of cracks [18,19]. This crack formation process likely occurs at high temperatures corresponding to the largest thermal expansion of the entire composite.

Figure 9a shows that the irreversible elongation of the B1 sample occurs after the first thermal cycle, which significantly differs from the results obtained for the A1 and A2 laminates (here material shrinkage occurred under all conditions). This elongation is caused by the release of the generated pressure stresses and related strains that was characterized by the large flat maximum in the temperature range from 100 °C to 240 °C. This maximum represents a superposition of the basic thermal expansion of Cu holes/laminate composite and release of the residual compressive strain.

The obtained data reveal that the post-curing process is suppressed during the first thermal cycle depicted in Figure 9. It is well known from the literature that both the curing and post-curing processes are suppressed due to high press [20]. In this work, samples of the B type were pressed several times and drilled to obtain Cu plated holes. This manufacturing pressing creates the residual compressive stress in the FR4 laminate structure, which inhibits the post-curing process. Higher pressures decrease the free volume between the chains of epoxy resin and thus reduce their mobility; as a result, the reaction freezes at low conversion. After releasing the pressure, the free volume recovers due to volume relaxation. Hence, relaxation of the compression residual deformation at high temperatures increases the magnitude of CTE up to 280 × 10^−6^ K^−1^ at 180 °C, which can promote cracks formation in the Cu-plated holes.

The post-curing process occurring during the second thermal cycle (Figure 9b) proceeds to a larger extent due to the partial removal of the residual strain in the first run. A lower CTE is obtained after the second cycle; however, the temperature dependence of CTE above the post-curing temperature range exhibits some curvature, suggesting that not all compressive residual strain was released in the first run and that the post-curing process may occur during the subsequent heating. Hence, the compressive residual stress increases the tensile stress of Cu in the Cu-plated hole even during the second thermal cycle.

Figure 10 shows the results obtained for the B2 sample. In this figure, the temperature dependences of the dimensional changes and CTE determined for the first thermal cycle of the B1 sample are shown as well, which can be used to characterize the transformation of the composite Cu-plated hole/FR4 laminate in the Mistral 260 oven. The first heating of sample B2 occurred inside the oven suggesting that the residual strain was partially removed before TMA measurements. The CTE value obtained after the first thermal cycle is lower than that of the B1 sample determined after one run followed by the post-curing process. The latter caused irreversible sample shrinkage after this thermal cycle (in contrast to the B1 sample which exhibited elongation after the first thermal cycle). The results obtained for the B2 sample in the first measurement cycle also show that the complete elimination of the residual strain inside the oven did not occur because its further decrease was observed during following thermal cycle. 

**Figure 11:**
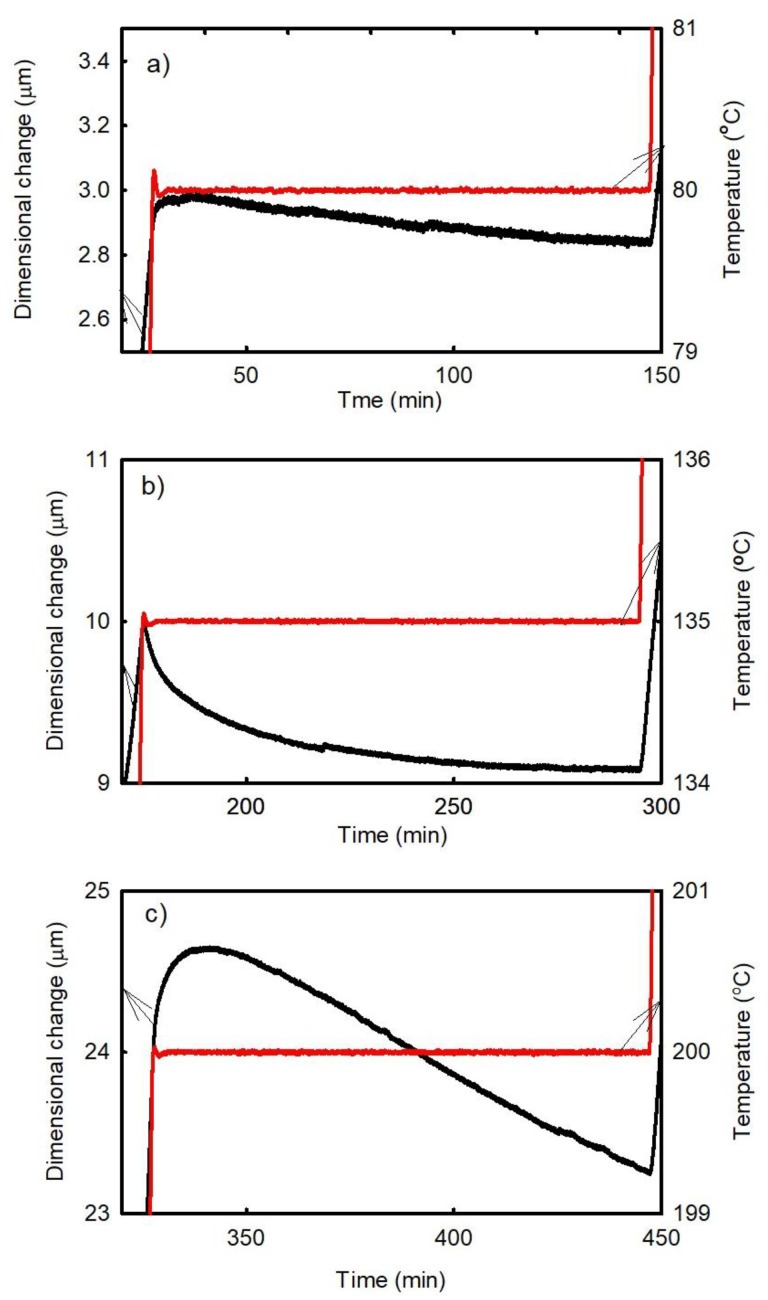
Time dependences of the dimensional changes of the B1 sample (FR4 laminate/Cu plated holes) observed at (**a**) 80 °C, (**b**) 135 °C, and (**c**) 200 °C.

The time dependences of the dimensional changes of the B1 sample observed at temperatures of 80 °C, 135 °C, and 200 °C are presented in Figure 11. Figure 11a shows that small sample shrinkage occurs at low temperatures corresponding to the rigid state of the laminate. Figure 11b displays the decrease in the dimensional changes observed during the post-curing process. Finally, Figure 11c shows the results obtained in the high temperature range, in which both processes (the release of the compression residual strain and sample shrinkage due to the viscous nature of the material) occur simultaneously. In the first part of the temperature dependence of dimensional changes, the elongation due to the relaxation of the residual strain dominates (this process is also time-dependent).

It should be noted that all measurements conducted for the samples of type B characterize the dimensional changes occurring inside the Cu-plated hole. Outside the hole, their values are different. The thermal and residual stresses generated in the B-types samples are localized at the interface between the Cu layer and FR4 laminate with the largest difference between the corresponding CTE values.

## 4. Conclusions

Dimensional changes of the FR4 laminate and FR4 laminate/Cu plated hole samples in the z-direction were determined by TMA in the temperature range from RT to 240 °C. During measurements conducted for the FR4/Cu plated hole specimens, the TMA probe was placed directly on the Cu hole. The manufacturing of both materials involved the pressing step, which resulted in the relaxation of the compression residual stress during heating and related elongation of the samples after the thermal cycle. Imperfect curing during manufacturing is another reason for irreversible dimensional changes. Thus, the irreversible shrinkage of pure FR4 laminate was observed in the temperature range from 110 °C to 150 °C. The post-curing process in the as-prepared samples proceeded to a larger extent than in the samples treated inside the oven. Furthermore, the relaxation of the compression residual strain occurs at temperatures higher than that of the post-curing process. While its effect on the dimensional changes of pure FR4 laminate is relatively small, it is substantial for the FR4 laminate/Cu plated holes (in the case of the as-prepared material, it even leads to sample elongation).

In the Cu part of the FR4/Cu plated hole composite, tensile stress was generated in the entire temperature range. Its magnitude increased with the release of the compression residual strain. While the CTE of the as-prepared specimen measured after the second thermal cycle at 170 °C was about 120 × 10^−6^ K^−1^, it reached a value of 240 × 10^−6^ K^−1^ after the first thermal cycle, which increased the possibility of crack formation in the Cu hole.

The compression residual stress generated in the as-prepared FR4/Cu plated holes during manufacture strongly suppressed the post-curing process, which occurred only to a relatively small extent. The extent of post-curing process increased in the second run after relaxation of the compression residual strain during the first run. Moreover, the higher pressure applied to the material decreased the free volume between the chains of epoxy resin, which significantly reduced their mobility; as a result, the reaction froze at low conversion. After releasing the pressure, the free volume was recovered (volume relaxation), and the post-curing process occurred. The latter can be observed only in the limited temperature range from 100 °C to 150 °C. Similar to other basic thermal expansion processes, the post-curing process and relaxation of the compression residual stress are time-dependent.

## Figures and Tables

**Table 1:** CTE values obtained for the Cu, A1, and B1 samples after 2 runs.

Sample	CTE (10^−6^ K^−1^)	
	100 °C	200 °C
Cu	17.2	17.8
A1	47.6	264.2
B1	42.5	173.2
